# Coexistence of blaNDM and blaOXA-48 Genes in Carbapenem-Resistant Burkholderia pseudomallei Isolated From Pus: A Rare Phenomenon

**DOI:** 10.7759/cureus.50671

**Published:** 2023-12-17

**Authors:** Gaurav Verma, Nipa Singh, Ambika Mohanty, A. Raj Kumar Patro, Dipti Pattnaik

**Affiliations:** 1 Department of Microbiology, Kalinga Institute of Medical Sciences, Bhubaneswar, IND; 2 Department of General Medicine, Kalinga Institute of Medical Sciences, Bhubaneswar, IND; 3 Department of Molecular Biology, Kalinga Institute of Medical Sciences, Bhubaneswar, IND

**Keywords:** melioidosis, ndm, oxa - 48, carbapenem resistant, burkholderia pseudomallei

## Abstract

*Burkholderia pseudomallei* causes melioidosis in both humans as well as animals and is classified as a tier 1 pathogen by the US CDC. Melioidosis is a disease that occurs predominantly in subtropical and tropical regions. It is endemic to northern Australia and parts of Southeast Asia, as well as the Indian subcontinent. Diagnosis can be made through history, clinical examination, imaging, and microbiological studies. We report a case where *Burkholderia pseudomallei *was isolated froma 41-year-old man who complained of pain in the left hip and the left shoulder and swelling in both lower limbs. Chest X-ray showed bilateral consolidation. USG of the left shoulder and bilateral hips showed a mass in the anterior region of the left upper arm and the lateral region of the left thigh. Pus aspirated from left shoulder grew *Burkholderia pseudomallei* on culture and was carbapenem-resistant. The isolate harbored two carbapenemase genes, blaNDM and blaOXA-48,* *which is a novel finding.

## Introduction

*Burkholderia pseudomallei* is the causative agent of melioidosis. Originally described by Whitmore and Krishnaswami in 1912 after reports of severe pneumonia and septicemia in Burma, the first case of melioidosis in Australia was described in 1950 in a patient with diabetes who died of septic melioidosis in Townsville [1]. The majority of India's population lives in rural areas and can easily become infected through direct contact with soil and water. Melioidosis can present with diverse clinical manifestations, including pneumonia, genitourinary infection, skin and soft tissue infection, internal organ abscesses, septic arthritis, neurological melioidosis, and fulminant septicemia without evident focus [2]. South Asia accounts for more than 44% of the cases reported worldwide, with India being a "hotspot" for the disease as the majority of India’s population lives in rural areas. The prevalence of melioidosis in India is widespread, with Maharashtra, Tamil Nadu, Orissa, Tripura, Kerala, West Bengal, and North Eastern states being the most affected regions. In India, the majority of cases were reported from coastal areas, with males being most commonly affected [3]. In 76% of the cases, the presence of an underlying disease is a crucial risk factor for melioidosis, with diabetes mellitus having a relative risk of 100-fold [4]. The first reported case of melioidosis in India was from Mumbai [5]. Reports regarding carbapenem resistance in *Burkholderia pseudomallei* are few, and in India, the first such case reporting the presence of a carbapenemase encoding blaOXA-57 gene was published in 2019 [6].

Here, we report a case of *Burkholderia pseudomallei* showing resistance to meropenem and harboring two carbapenemase encoding genes, blaNDM and blaOXA-48, which is a rare phenomenon.

## Case presentation

**Figure 1:**
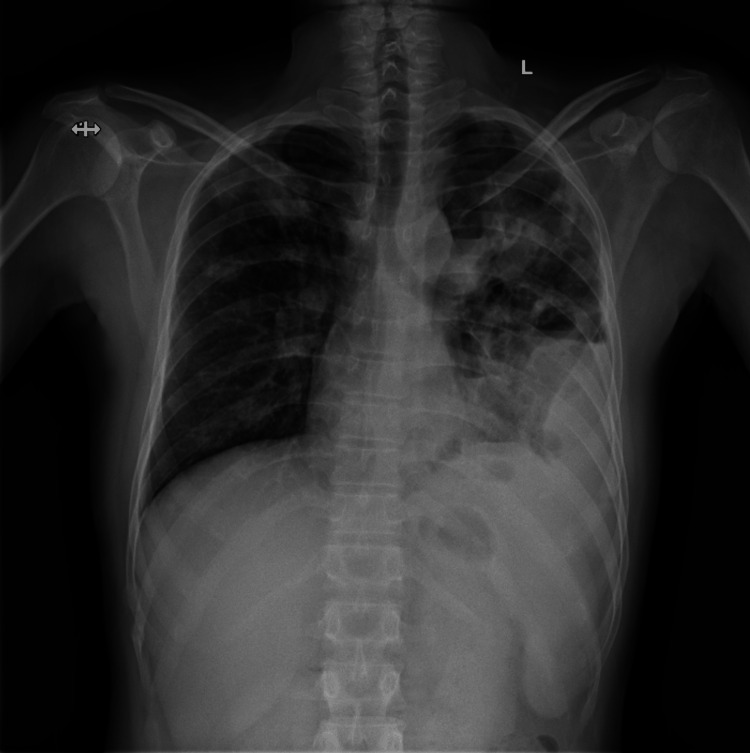
Chest X-ray shows bilateral consolidation

A 41-year-old male, a farmer by occupation, belonging to a rural area was admitted to a tertiary care hospital in Bhubaneswar, India, with the chief complaints of pain in the left hip for 15 days, pain in the left shoulder for eight days, and swelling in both lower limbs since four days. The pain increased on walking and on raising the upper limb. The patient was a known case of diabetes mellitus on human insulin. On admission, a general examination revealed a pulse rate of 132/minute, blood pressure of 110/70 mmHg, respiratory rate of 20/minute, and temperature of 98°F. Relevant investigations were sent and the patient was started on antibiotics (cefuroxime 500 mg one tablet twice a day (BD), sulfasalazine 500 mg one tablet once a day (OD), prednisone 20 mg one tablet OD, etoricoxib 90 mg one tablet OD, and injection H. Actrapid 10U BBF, 10 BL, 8U BD S/C). On admission, hematological investigations were as follows: WBC count of 19.3 X 10³/ul, RBC count of 3.19 X10⁶/ul, hemoglobin of 7.4 gm/dl, packed cell volume of 24.6%, mean corpuscular volume of 77%, mean corpuscular hemoglobin of 23.2 pg, and erythrocyte sedimentation rate of 120 mm. On chest X-ray (posteroanterior view) bilateral consolidation was seen (Figure 1).

**Figure 2:**
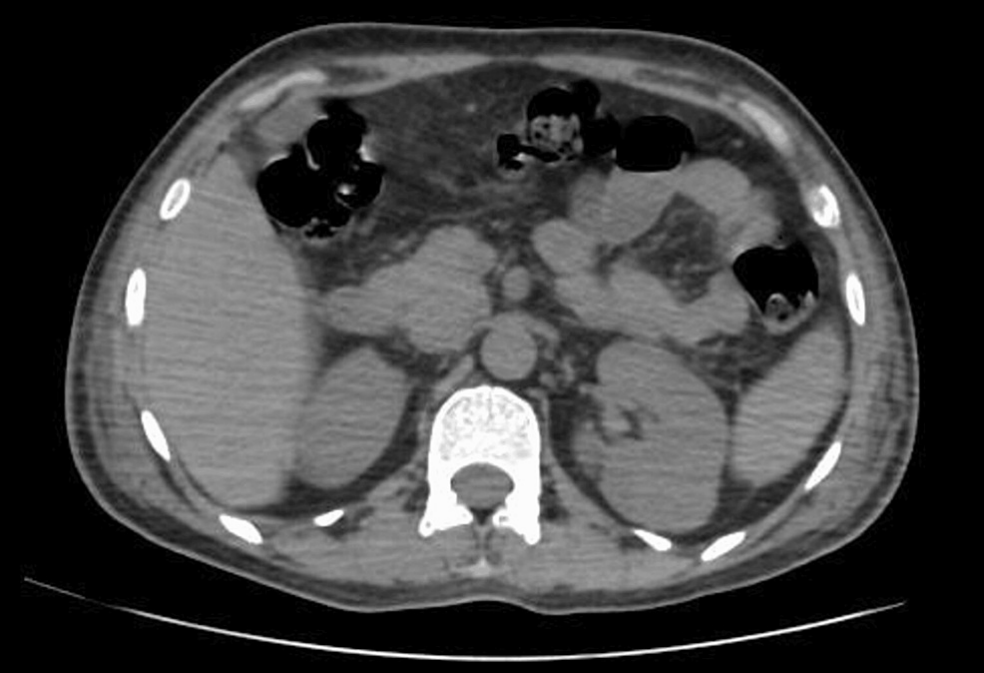
Contrast-enhanced computed tomography of the whole abdomen

USG of the left shoulder and bilateral hip showed a collection in the anterior aspect of the left upper arm and lateral aspect of the left upper thigh, suggestive of an infective abscess. Contrast-enhanced computed tomography of the whole abdomen showed splenomegaly with tiny non-enhancing foci/granuloma/abscess and encysted intra-muscular collection along the lateral aspect of left gluteus medius muscle, bilateral pleural effusion with multiple cavitatory nodules in both lungs and patchy consolidation, suggestive of infective or neoplastic origin (Figure 2).

Bilateral lower limb venous Doppler showed phlebitis, left common femoral vein, and right great saphenous vein thrombosis. An orthopedic consultation was taken, pus was aspirated from the left shoulder, 50-60 ml pus was drained, and it was sent for culture. Aerobic, motile, Gram-negative bacilli with bipolar staining in pure culture, was identified as *Burkholderia pseudomallei* (99% probability) by the Vitek-2 AST system (bioMérieux, Marcy-l’Etoile, France). Antimicrobial susceptibility antibiotics were determined by Vitek-2 AST and interpreted as per Clinical and Laboratory Standards Institute (CLSI) guidelines [7]. The isolate was resistant to ceftazidime and meropenem and sensitive to cotrimoxazole (Table 1).

**Table 1:** Antibiograms of blaNDM and blaOXA-48-positive Burkholderia pseudomallei

Antibiotic(s)	Susceptibility
Piperacillin/tazobactam	R
Ceftazidime	R
Cefoperazone/sulbactam	R
Cefepime	I
Imipenem	R
Meropenem	R
Amikacin	R
Gentamicin	R
Ciprofloxacin	I
Minocycline	S
Tigecycline	R
Trimethoprim/sulfamethoxazole	S
Aztreonam	R
Levofloxacin	R
Ofloxacin	R
Doxycycline	S
Chloramphenicol	S

Although resistance compromises the therapeutic efficacy of the commonly used treatment regimens, in our case, clinical improvement was observed after the initiation of therapy of meropenem and levofloxacin [8]. Hence, tablet faropenem and levofloxacin were continued despite in vitro resistance to meropenem and levofloxacin. The patient was successfully treated with meropenem for a period of 16 days followed by tablet levofloxacin and tablet faropenem for a period of eight days during the hospital stay, and on discharge, he was advised to continue with tablet levofloxacin and tablet faropenem for a further period of seven days and tablet Bactrim DS (sulfamethoxazole and trimethoprim) for three months.

The patient recovered well and was discharged from the hospital. The patient has reported to the OPD for follow-up after discharge from the hospital. He has completed the medication advised and is clinically and hemodynamically stable.

The isolate was subjected to sequencing (using 16S ribosomal RNA gene). The obtained chromatogram was analyzed with the available sequence in the National Center for Biotechnology Information (NCBI) database and the sequence result confirmed the findings of *B. pseudomallei*.

**Figure 3:**
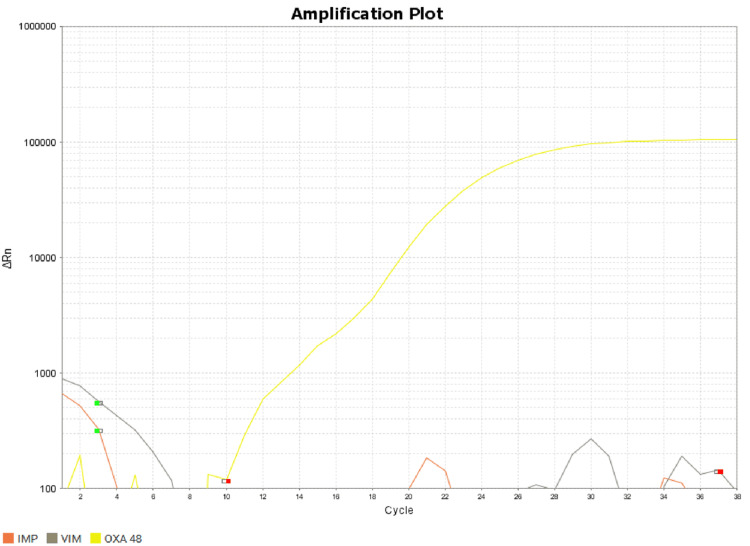
Real-time polymerase chain reaction amplification of sample showing blaOXA-48 gene in B. pseudomallei OXA-48: yellow; IMP: red; VIM: grey.

**Figure 4:**
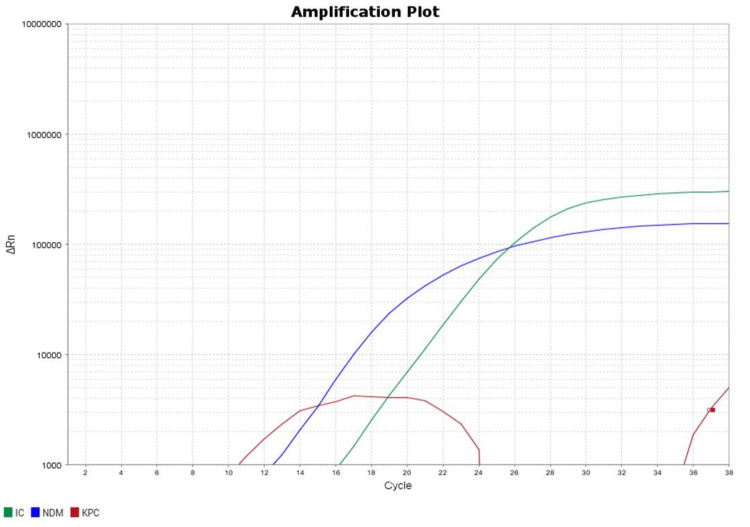
Real-time polymerase chain reaction amplification of the sample showing the blaNDM gene in B. pseudomallei IC: internal control (green); NDM (blue); KPC (red).

As the isolate was resistant to meropenem, molecular detection to detect carbapenemase genes was carried out using real-time polymerase chain reaction (PCR). In brief, DNA extraction of the isolate was done (by using a TRUPCR Total Nucleic Acid Extraction Kit, 3B BlackBio Biotech India Ltd., New Delhi, India), and reverse transcription-polymerase chain reaction (RT-PCR) was done to detect the blaNDM, blaOXA-48, blaKPC, blaIMP, and blaVIM genes (by Carbapenem Resistance Detection Kit version 1.0, 3B BlackBio Biotech India Ltd., New Delhi, India) as per manufacturer’s instruction. Co-occurrence of blaNDM and blaOXA-48 genes was noted (Figures 3, 4).

## Discussion

*B. pseudomallei* is the causative agent of melioidosis. The endemic cases of melioidosis are seen mainly in certain parts of Asia and Australia, with certain instances reported in Africa, the Caribbean, and the Middle East [9]. *B. pseudomallei* is a highly adaptable organism, capable of producing a range of clinical manifestations depending on the infected tissue and maintaining a survival advantage in infected hosts and environments [10]. Inhalation, ingestion, and inoculation are well-known modes of acquisition of melioidosis. It is extremely rare for *B. pseudomallei* to be transmitted from person to person and sexual transmission, in particular, has been proposed but has yet to be proven as a mode of infection [11]. The majority of people who have been exposed to *B. pseudomallei* show no signs or symptoms; those who demonstrate symptoms range from minor, such as fever and skin problems, to severe, such as pneumonia, abscesses, and septic shock, which can lead to death [12]. The most common risk factors for melioidosis have been identified as renal disease, chronic lung disease, diabetes, and alcohol consumption [9].

People of all ages can develop human melioidosis, but it is most common in adults between 40 and 60 years old. The severity and occurrence of melioidosis depend on various factors, such as the host, environment, and specific virulence factors of the strain [12,13]. In this study, the presence of both blaNDM and blaOXA-48 was detected in *B. pseudomallei*. In a study in south India by Amladi et al., blaOXA-57 carrying *B. pseudomallei* was reported, while Keith et al. identified OXA-57 in clinical and environmental isolates of *B. pseudomallei* and *Burkholderia thailandensis*. A study by Marutpong et al. shows that all clinical isolates of *B. pseudomallei* harbored blaOXA [6,14,15]. It is advisable to monitor the antibiotic resistance genes to keep a record of the prevailing pattern in a geographical area.

*B. pseudomallei* is intrinsically resistant to a wide range of antibiotics and hence the treatment options for melioidosis are limited. Treatment of melioidosis involves an intensive phase and an oral eradication phase. The intensive phase includes intravenous ceftazidime or meropenem and either trimethoprim-sulfamethoxazole, doxycycline, or amoxicillin-clavulanic acid can be used in eradication therapy [8].

Carbapenems remain the mainstay of treatment against *B. pseudomallei* and resistance to carbapenems will be a challenge in the treatment of melioidosis. Meropenem resistance in *B. pseudomallei* has been reported in a patient from Australia but no carbapenemase genes could be detected in this case [16]. A literature search across all platforms did not reveal any reports of the coexistence of blaNDM and blaOXA-48 in *B. pseudomallei*.

## Conclusions

The isolate found in our study was resistant to ceftazidime and carbapenem but sensitive to co-trimoxazole. The presence of blaNDM and blaOXA-48 in *B. pseudomallei* poses a serious threat as they exhibit increased resistance to antibiotics. Carbapenem resistance poses a significant risk of treatment failure for hospitalized patients. Early detection in a well-equipped microbiological laboratory combined with high clinical suspicion and appropriate infection control measures are essential to combat infection and its spread. To the best of our knowledge, there are no published reports of the coexistence of OXA-48 and NDM genes in *B. pseudomallei*.
